# *Manilkara zapota* (Linn.) Seeds: A Potential Source of Natural Gum

**DOI:** 10.1155/2014/647174

**Published:** 2014-03-04

**Authors:** Sudarshan Singh, Sunil B. Bothara

**Affiliations:** ^1^H.S.B.P.V.T. Group of Institutions, College of Pharmacy, Kashti, Ahmednagar, Maharashtra 414701, India; ^2^Bhagwan College of Pharmacy, Aurangabad, Maharashtra 431001, India

## Abstract

Mucilage isolated from seeds of *Manilkara zapota* (Linn.) P. Royen syn. is a plant growing naturally in the forests of India. This mucilage is yet to be commercially exploited, and characterized as polymer. Various physicochemical methods like particle size analysis, scanning electron microscopy, thermal analysis, gel permeation chromatography, X-ray diffraction spectrometry, zeta potential, Fourier transform infrared spectroscopy, and nuclear magnetic resonance spectroscopy have been employed to characterize this gum in the present study. Particle size analyses suggest that mucilage has particle size in nanometer. Scanning electron microscopy analysis suggests that the mucilage has irregular particle size. The glass transition temperature of the gum was observed to be 138°C and 136°C by differential scanning calorimetry and differential thermal analysis, respectively. The thermogravimetric analysis suggested that mucilage had good thermal stability. The average molecular weight of mucilage was determined to be 379180, by gel permeation chromatography, while the viscosity of mucilage was observed to be 219.1 cP. The X-ray diffraction spectrometry pattern of the mucilage indicates a completely amorphous structure. Elemental analysis of the gum revealed the contents of carbon, hydrogen, nitrogen, and sulfur to be 80.9 (%), 10.1 (%), 1.58 (%), and 512 (mg/kg), respectively. Mucilage had specific content of calcium, magnesium, potassium, lower concentrations of aluminum, cadmium, cobalt, lead, and nickel. The major functional groups identified from FT-IR spectrum include 3441 cm^−1^ (–OH), 1660 cm^−1^ (Alkenyl C–H & C=C Stretch), 1632 cm^−1^ (–COO–), 1414 cm^−1^ (–COO–), and 1219 cm^−1^ (–CH_3_CO). Analysis of mucilage by paper chromatography and 1D NMR, indicated the presence of rhamnose, xylose, arabinose, mannose, and fructose.

## 1. Introduction

In recent years, plant derived polymers have evoked tremendous interest due to their diverse pharmaceutical applications such as diluents, binders, disintegrants in tablets, thickeners in oral liquids, protective colloids in suspensions, gelling agents in gels, and bases in suppository [1]; they are also used in cosmetics, textiles, paints, and paper-making [2].

The plant based polymers have been studied for their application in different pharmaceutical dosage forms like matrix controlled system, film coating agents, buccal films, microspheres, nanoparticles, and viscous liquid formulations like ophthalmic solutions, suspensions, and implants and their applicability and efficacy has been proven [3–5]. These have also been utilized as viscosity enhancers, stabilizers, disintegrants, solubilizers, emulsifiers, suspending agents, gelling agents and bioadhesives, and binders in the above mentioned dosage forms [6].

The term “mucilage in plants” means those substances which are soluble or at least swell very perceptibly in water and which, upon the addition of alcohol, are precipitated in a more or less amorphous or granular mass. Mucilage originates in the plant either as a part of the contents of the cell or as a part of the wall thereof.

Many natural polymeric materials have been successfully used in sustained-release tablets. These materials include guar gum, ispaghula husk, galactomannan from *Mimosa scabrella*, *Gleditsia triacanthos Linn* (honey locust gum), *Sesbania* gum, mucilage from the pods of *Hibiscus esculenta*, tamarind seed gum, gum copal and gum dammar, agar, konjac, and chitosan [7]. Industrial gums and mucilage, which, for the most part, are water-soluble polysaccharides, have enormously large and broad applications in both food and non-food industries. Their use depends on the unique physicochemical properties that they provide, often at costs below those of synthetic polymers.

*Manilkara zapota* (Linn.) P. Royen syn. is a large, evergreen, forest tree more than 30 m in height a tree belonging to the family Sapotaceae. Earlier, toxicological evaluation of seed mucilage of *Manilkara zapota* (Linn.) P. Royen syn was carried out so as to enable its use as a food additive [8]. An understanding of the physicochemical properties and structural characterization of mucilage is essential in exploiting its potential as a food additive and for other industrial applications.

In the present paper, we report morphological, physicochemical and structural aspects of mucilage from seeds of *Manilkara zapota* (Linn.) P. Royen syn., in order to provide a separate identity to this tree gum. The methods employed for the analysis include (i) particle size analysis by Microtrac, (ii) scanning electron microscopy (SEM), (iii) gel permeation chromatography (GPC), (iv) differential scanning calorimetry (DSC), (v) differential thermal analysis (DTA), (vi) thermogravimetry analysis (TGA), (vii) zeta potential (ZP), (viii) elemental analysis (carbon (C), hydrogen (H), nitrogen (N), and sulfur (S)), (ix) mineral analysis, (x) powder X-ray diffraction spectrometry (PXRD), (xi) Fourier transform infrared spectroscopy (FT-IR), (xii) 1D nuclear magnetic resonance (NMR), and (xiii) paper chromatography analysis.

## 2. Materials and Methods

### 2.1. Materials

Fruits of *Manilkara zapota* were collected from the forest of Rajgamar, District Korba, Chhattisgarh, in the months of April–June and authenticated by Professor H. B. Singh, NISCAIR, New Delhi, India. Mucilage was isolated from the seeds using maceration techniques in which seed powder (100 g) was soaked in cold distilled water (500 mL) and slurry was prepared. Then slurry was kept aside for a day, then solution was heated on Bunsen burner for 1 h; after one day the mixture was filtered with the help of muslin cloth. The filtrate was centrifuged (Remi) at 3000 rpm for 10 minutes. The supernatant was collected after centrifugation then double volume of acetone was added in it to precipitate the mucilage. The precipitate was washed with chloroform. The mucilage was then dried at 40°C–45°C in hot-air oven (BioTech) and then passed through mesh number 120 and stored in desiccators until used for further studies in powder form [9, 10]. Deionized water was used for all experiments. All other chemicals used were of analytical grade.

### 2.2. Determination of Particle Size by Particle Size Analyzer

Particle size was determined by particle size analyzer (Zetatrac, Microtrac). Dispersion of sample was prepared in suitable solvent (water and 0.1 N NaCl) and the relevant information such as density, viscosity, and dielectric constant was added for the solvent in software (Microtrac FLEX Operating Software). About 3.0 mL sample dispersion was added in sample holder. Sample holder made of optical probes which are paired with their opposite electrodes in an insulating sample cell. An electric field is applied between the optical probes and their corresponding electrodes. Particle in motion analyzed under the influence of electric field. Particle size distribution was determined from the velocity distribution of particles suspended in a dispersing medium, using the principles of dynamic light scattering [11]. The above procedure was performed in triplicate.

### 2.3. Scanning Electron Microscopy

Microphotograph of mucilage was recorded by using scanning electron microscopy (Philips). Appropriate samples were mounted on an aluminum stub with double-sided adhesive tape. The tape was first firmly attached to the stub and the sample powder was scattered carefully over its surface. The stub with the sample was then coated with a thin layer of gold to make the sample conductive. The processed specimen was subjected to SEM analysis [12].

### 2.4. Molecular Weight by Gel Permeation Chromatography

GPC is a technique which enables polymer molecules of different sizes in solution to be separated from one another, such that the large molecules elute first and small molecules elute last. As they emerge from the bottom of the column, they are detected by a differential refractometer. Since the molecular size in solution (hydrodynamic volume) is related to *M*_*w*_ (molecular weight), a picture of the entire *M*_*w*_ distribution is obtained quickly and simply [13].

GPC was carried out to estimate molecular weight of the mucilage relative to dextran polysaccharide as standard, using Waters Alliance model (Waters 2695 separation module with autoinjector) coupled with Waters 2414 refractive index detector (RI). Mobile phase was 0.2 M NaNO_3_ in water, flow rate was 1.0 mL/min, ultrahydrogel 500 and ultrahydrogel 120 (7.8 mm × 30 cm × 9 um) were in series. Detector and column were operated at 30°C, which was started from *M*_*w*_: 5,200; 48,600; 2,03,000; 6,68,000; 14,00,000 spectra were processed using Empower Software.

### 2.5. Differential Scanning Calorimetry

DSC analysis for mucilages was performed using a differential scanning calorimeter (Mettler Toledo Star System). Accurately weighed (5 mg) samples were placed into platinum cups and sealed. The temperature range was from 0°C to 300°C under nitrogen atmosphere at a heating rate of 10°C/min [14].

### 2.6. Differential Thermal Analysis

DTA is a very popular thermal analysis technique; it measures endothermic and exothermic transitions as a function of temperature. The analysis mucilage was performed on differential thermal analysis (Stapt, Linseis) in which accurately weighed (5 mg) samples were placed into platinum cups and sealed. The temperature range was from 0°C to 240°C at a heating rate of 15°C/min [15].

### 2.7. Thermogravimetry Analysis

TGA is a thermal analysis technique for measuring the mass variation in a sample as a function of temperature under a controlled atmosphere. This variation of mass can be positive (mass gain) when the sample is subjected to oxidation or corrosion, or else negative (mass loss) when part of the sample has been transformed into vapor. Thermal stability behavior can be investigated in both conditions: (1) dynamic, in which the temperature is increased at a linear rate and (2) isothermal, in which the temperature is kept constant [15].

The analysis of mucilages was done using thermal analyzer (Stapt, Linseis, Germany) in which accurately weighed (15 mg) samples were placed into platinum cups and sealed. The temperature range was from 0 to 900°C at a heating rate of 15°C/min.

### 2.8. Electrokinetic Studies: Zeta Potential

ZP is related to the charge present on the surface or near-surface of a suspended particle. ZP was determined using Zetatrac (Microtrac) by measuring the response of charged particles to an electric field. In a constant electric field particles float at a constant velocity. Through this velocity and charge ZP can be determined. Zetatrac utilizes a high frequency AC electric field to oscillate the charged particles. The Brownian motion power spectrum is analyzed with the nanotrac controlled reference technique of particle sizing to determine the modulated power spectrum (MPS). This is a component of the power spectrum resulting from the oscillating particles. ZP was calculated for mucilage from the MPS signal using formula [16] in water and pH dependence of the zeta potential was investigated with the background electrolyte of 0.1 N NaCl

(1) $$\begin{array}{c} \zeta =\frac{\mu \eta }{\varepsilon }, \end{array}$$

where *ζ* is zeta potential, *μ* is mobility, *η* is viscosity, and *ε* is dielectric constant for water at 25°C, zeta potential (mV) ~ 12.8 × Mobility (*μ*/sec/volt/cm).

### 2.9. Elemental Composition (CHNS) and Heavy Metal Analysis of Mucilage

Elemental compositions (CHNS) were analyzed using elemental analyzer (Elemental, Vario). Accurately weighed 0.5 g of sample was heated to 1150°C and corresponding element was determined by using elemental analyzer [17].

### 2.10. Powder X-Ray Diffraction Pattern

PXRD patterns of mucilage were recorded using X-ray diffractometer (Goniometer). The experiments were carried out at 25°C under the following conditions: voltage 40 Kv, current 30 mA, and 2*θ* angle with scan step time of 10.33 s with specific length of 10 mm [15].

### 2.11. Fourier Transform Analysis

FTIR spectra of mucilage were recorded on a FT-IR spectrometer (Thermo Scientific). The dry powder was mixed with KBr and pressed into pellets under mechanical pressure. The FT-IR spectra were obtained by scanning between 4000 and 400/cm [18].

### 2.12. 1D Nuclear Magnetic Resonance

NMR spectra of ^1^H and ^13^C of mucilage were recorded in an NMR (400 MHz) spectrometer (Bruker Avance II 400). The test mucoadhesive agent (100 mg) was dissolved in D_2_O and chemical shifts were reported in ppm relative to an internal standard TSP (3-trimethylsilylpropionic-2,2,3,3,-d4 acid, sodium salt, 98% D) for ^1^H NMR and 1,4-dioxane (d 66.67 ppm) for ^13^C spectra. Proton NMR spectra were obtained at a base frequency of 400 MHz, with 16 transitions and delay time 1.5 s and for ^13^C, the base frequency was 100 MHz, with 3000 scans and delay time 2 s [19].

### 2.13. Sugar Composition by Paper Chromatography

Carbohydrate or sugars occupy a central position in plant metabolism, so the method of their detection and estimation is very important. Sugars are conveniently classified into three groups, on the basis of molecular size: the simple monosaccharide's (e.g., glucose and fructose) and their derivatives, the oligosaccharides, by the condensation of two or more monosaccharide's units (e.g., sucrose), and the polysaccharides which consist of long chains of monosaccharide's units, joined head to tail, either as straight chains or without branching [20]. The samples were prepared in distilled water for paper chromatography. The paper chromatography analysis was performed using n-butanol: acetic acid: water (4 : 1 : 1) as solvent system with using arabinose, fructose, mannose, rhamnose, and xylose as reference standards.

## 3. Results

### 3.1. Particle Size Analysis

The average particle size as determined by Zetatrac particle size analyzer indicated that obtained mucilages were in fine particle size and average range was obtained as 500–551 nm diameters.

### 3.2. Scanning Electron Microscopy

**Figure 1:**
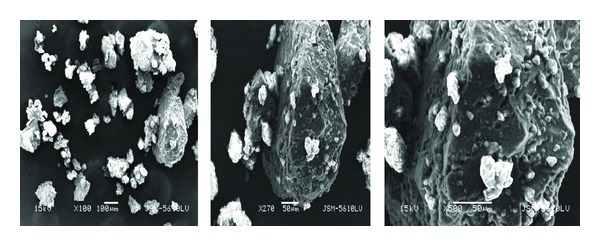
Scanning electron microscopy of mucilage at different magnifications using SEM.

SEM of mucilage obtained is represented in Figure 1 at different magnifications. The microphotographs of mucilages are indicative of an amorphous material. The particles are mostly seen as aggregates of irregular shapes and dimensions which were fibrous in nature.

### 3.3. Molecular Weight by Gel Permeation Chromatography

The molecular weight of mucilages were determined by gel permeation chromatography and expressed as the “Dextran polysaccharide equivalent” molecular weight. The computed average molecular weights, (*M*_*w*_), number of average molecular weight, (*M*_*n*_), and polydispersity (*M*_*w*_/*M*_*n*_) are tabulated in Table 1.

### 3.4. Differential Scanning Calorimetry

**Figure 2:**
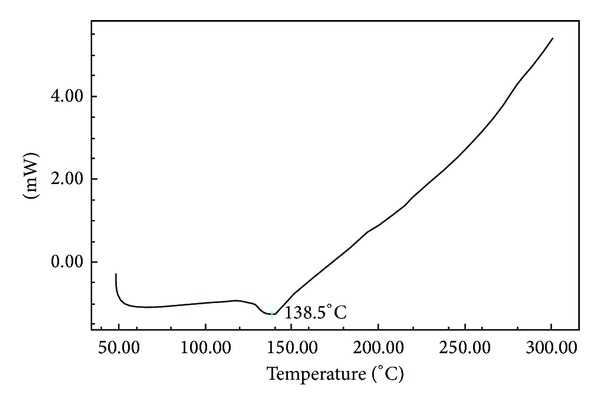
Differential scanning calorimetry (DSC) characterization of mucilage Using DSC analyzer.

DSC has emerged as powerful physical tools to monitor physical and chemical changes that occur in the gum during thermal processing and these methods yield curves that are unique for a given gum. The outcome of differential scanning calorimetry (DSC); analysis of mucilage revealed that the glass transition temperature is 138°C. The major intense peak recorded in the DSC thermograms is an endothermic transition (at around 200°C) followed by weaker exotherm(s). The DSC endotherm is presented in Figure 2.

### 3.5. Differential Thermal Analysis

**Figure 3:**
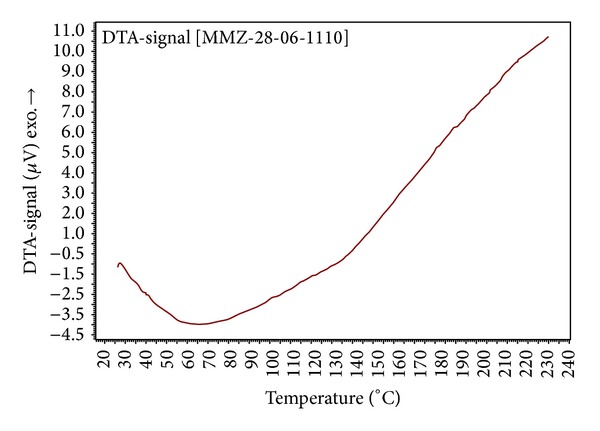
Differential thermal analysis (DTA) characterization of mucilage using DTA analyzer.

The result of differential thermal endotherm analysis is represented in Figure 3 for mucilage. The outcome of DTA analysis for mucilage reveals that the transition temperature is 136°C.

### 3.6. Thermogravimetry Analysis

**Figure 4:**
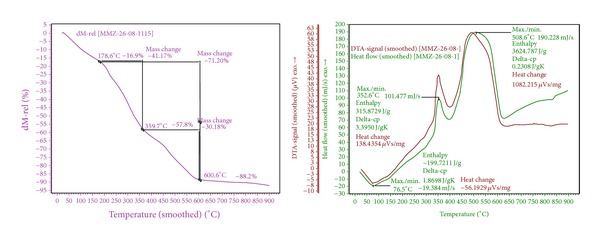
Thermogravimetry analysis characterization of mucilage using TGA analyzer.

TGA is a simple and accurate method for studying the decomposition pattern and the thermal stability of polymers.  Table 2 gives the details of thermal behavior according to the primary thermograms and derivative thermograms of mucilages. The representative plot results of thermogravimetric analysis carried out on the mucilages under lean oxygen (5% oxygen in nitrogen) atmosphere are shown in Figure 4. The details of thermal behavior and thermal stability data according to the primary thermograms and derivative thermograms for the gum showed that heating at a rate of 10°C per minute from 0°C to a maximum of 900°C resulted in two mass loss events.

### 3.7. Elemental Composition (CHNS) and Heavy Metal Analysis of Mucilage

The results of Elemental composition and Heavy Metal Characterization are tabulated in Table 3. The elemental analysis study showed that the isolated mucilage had certain percentage of carbon, nitrogen, sulphur, and hydrogen, while toxic heavy metal like arsenic, lead, cadmium, and mercury were absent.

### 3.8. Electrokinetic Studies (Zeta Potential)

The ZP measurement was performed to collect information on the stability and charge behavior of the polymer. The ZP of mucilage in aqueous medium (water) was recorded to be 18.05 mV and in 0.1 N NaCl was recorded to be 5.15 mV, respectively.

### 3.9. Powder X-Ray Diffraction Pattern

**Figure 5:**
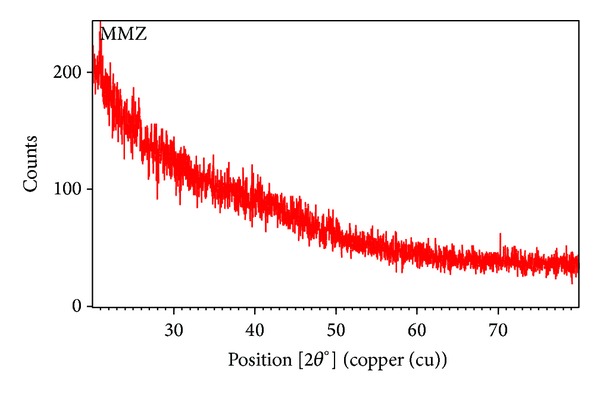
Powder X-ray diffraction spectra of mucilage obtained from seeds of* M. zapota* using goyenimeter.

PXRD analysis of mucilage is represented in Figure 5. The result indicated that no characteristic peaks were observed in the spectrum, indicating that the mucilages were completely amorphous in nature.

### 3.10. Fourier Transform Analysis

**Figure 6:**
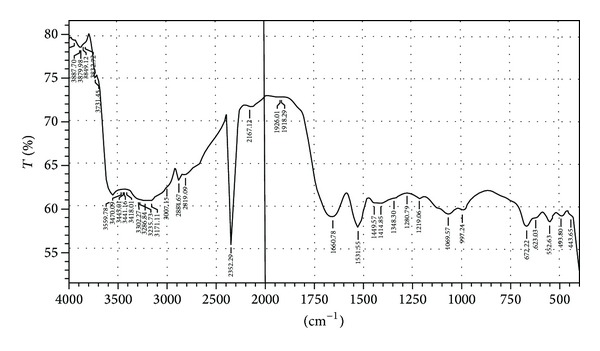
FTIR spectral characterization of mucilage using Thermo Scientific FTIR spectrophotometer.

The FT-IR spectrum of mucilage is presented in Figure 6. The interpretation of IR spectral analysis was done using “CHEMIX School Software.” Spectra exhibited the typical bands and peak characteristic of polysaccharides. The spectra of mucilage showed band occurring at 3559.78 cm^−1^ results from the presence of amide N–H & C=O stretch and amine N–H. The peak obtained at 3470.09–3418.01 cm^−1^ results from the presence of hydroxyl (–OH and –CO) groups. The peak obtained at 2884.67–2819.09 cm^−1^ results from stretching modes of the alkyl C–H stretch, carboxylic acid C=O & O–H stretch, and methylene (–CH_2_–) C–H stretch. The peak obtained at 2167.12–1660.78 cm^−1^ results from stretching mode of the alkenyl C–H & C=C stretch and amide N–H & C=O stretch.

### 3.11. 1D Nuclear Magnetic Resonance

**Figure 7:**
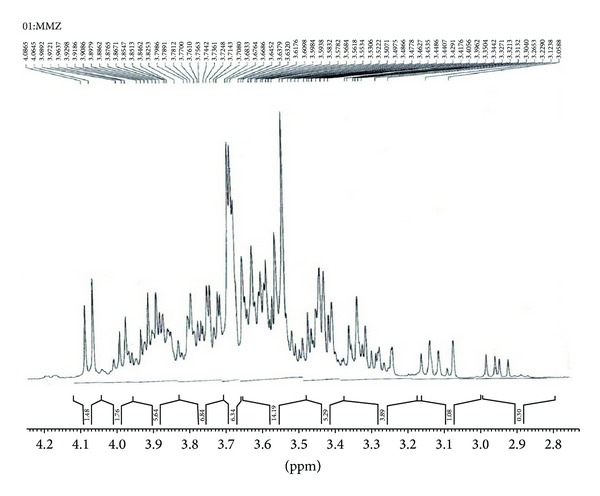
1D ^1^H NMR spectral characterization of mucilage using Bruker Avance II 400 NMR spectrophotometer.

**Figure 8:**
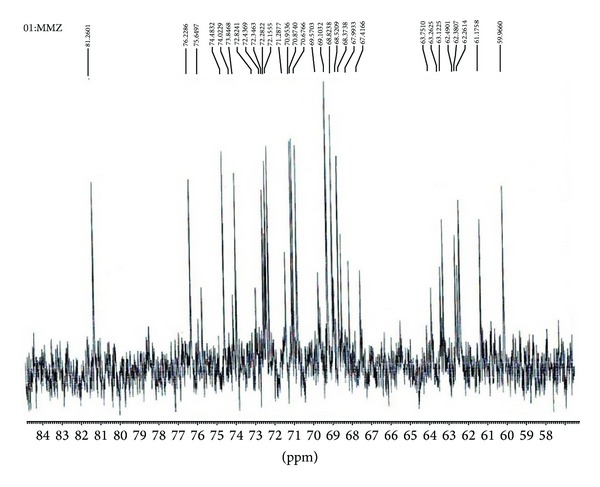
1D C^13^ NMR spectral characterization of mucilage using Bruker Avance II 400 NMR spectrophotometer.

The interpretation of ^1^H and C^13^ NMR spectra analysis was done using “CHEMIX School Software” and was represented in Figures 7 and 8. Spectra exhibit the typical bands and peak characteristic of polysaccharides. The ^1^H and C^13^ NMR spectra of mucilage indicated presence of certain sugar composition such as signals of ^1^H NMR spectra between d 3.58–d 3.40 ppm can be attributed to CH and OH group of rhamnose, mannose and arabinose. The signal at d 67.9 ppm, 72.2 ppm, and d 72.3 ppm of C^13^ NMR spectra can be attributed to CH group of rhamnose and mannose.

### 3.12. Sugar Composition by Paper Chromatography

The *R*_*f*_ value of standards arabinose, fructose, mannose, rhamnose, and xylose matched with the obtained *R*_*f*_ values of test substances (mucilage).

## 4. Discussion

The traditional concept of the excipients as any component other than the active substance has undergone a substantial evolution from an inert and cheaper vehicle to an essential constituent of the formulation. Excipients are any components other than the active substances intentionally added to formulation of a dosage form [21].

### 4.1. Particle Size Analysis

Micromeritics involve the study of small particles and the order of a few micron sizes. Particle size analysis involves the characterization of individual particles, particles size distribution, and powders. Particle size also previews the degree of densification, which could occur during tableting which was further also confirmed by SEM analysis.

### 4.2. Scanning Electron Microscopy

Microscopy is a major tool for the characterization of polymer material ultrastructure. In case of drug delivery system, microscopical examination enables an understanding of a number of variables that govern delivery. Such imaging technique is used to examine the detail shape, size, and distribution of polymeric micro- and nanoparticles, as well as interaction with biological environment [21]. The SEM results of the present study suggest that hydration capacity of mucilage depends on the surface property as presented in powder XRD studies. It was reported earlier that particle size and specific surface area influence the hydration behavior of gums, which in turn influence their intrinsic viscosity and molecular mass [22–24]. Further, Wang experimentally reported that particle size influenced the hydration kinetics and molecular mass of guar gum which is a galactomannan-rich tree gum [23].

### 4.3. Molecular Weight by Gel Permeation Chromatography

Polymer molecular weight determination is important because it determines many physical properties such as the temperatures for transitions from liquids to wax to rubber to solids and mechanical properties such as stiffness, strength, viscoelasticity, toughness, and viscosity. The polydispersity of isolated mucilage was obtained as 6.4. The polydispersity index (*M*_*w*_/*M*_*n*_) is used as a convenient measure of the range of molecular weight present in a distribution and is in the range of 1.5–7.0 for natural polysaccharide gums [24]. If the molecular weight is too low, the transition temperatures and the mechanical properties will generally be too low for the polymer material to have any useful commercial applications. For a polymer to be useful it must have transition temperatures to wax or liquids that are above room temperature and it must have mechanical properties sufficient to bear design loads. Further, molecular weight is also a physical property of compound, which helps in standardization.

### 4.4. Differential Scanning Calorimetry and Differential Thermal Analysis

Whenever a polymer undergoes a phase transition such as melting or a glass transition from the rigid “glass state” to the soft “rubber state,” the temperature trends to remain constant while energy is taken in to the system. DSC and DTA are essentially techniques that compare the difference between the energy acquired or released by a sample and a suitable reference as a function of temperature or time, while the sample and reference are subjected to a controlled temperature rise. The result of DSC and DTA revealed that the isolated mucilage from seeds has good stability. Dehydration, depolymerization, and pyrolytic decomposition are involved in DSC and DTA at high temperature stages and resulted in the formation of H_2_O, CO, and CH_4_. However, because of the difference in structures and functional groups, either the degradation routes or the resulting fragments will be different. The most of polysaccharides are comprised of carboxylate or carboxylic acid functional groups. Therefore, thermal scission of the carboxylate groups and evolution of CO_2_ from the corresponding carbohydrate backbone may be a probable mechanism for the thermal transitions. Accurate assigning of the thermal transitions is very difficult [16].

### 4.5. Thermogravimetry Analysis

Thermogravimetry analysis of mucilage showed two stages of decomposition. The early minor weight loss in samples is attributed to the loss of adsorbed and structural water of biopolymers as related by other authors [25, 26] or due to the desorption of moisture as hydrogen bound water to the saccharide structure. The second weight loss event may be attributed to the polysaccharide decomposition [27] and is described by a weight loss. The weight loss onset (representing the onset of oxidation or decomposition) of polymer suggests that mucilages have good thermal stability.

### 4.6. Elemental Composition (CHNS) and Heavy Metal Analysis

Elemental analysis is a process where a sample of some materials (e.g., soil, minerals, and chemical compounds) is analyzed for its elemental composition such as carbon, hydrogen, nitrogen, and sulphur. Elemental analysis can be qualitative (determining what elements are present), and even it can be quantitative. The elemental and heavy metal analysis showed presence of certain percentage of carbon, nitrogen, sulphur, and hydrogen and absence of heavy metals in mucilages. The present study indicated similar compositions of elements as reported earlier for gum kondagogu [17]. The presence of nitrogen may indicate the presence of protein in the sample. Further study also suggested the presence of S-containing protein and amino acids and also confirms the formation of bond (primary strong covalent bonds, weak secondary hydrogen bond, and Vander Waal's forces) of mucilage with mucosa in a short duration of time.

### 4.7. Electrokinetic Studies

The electrokinetic behavior depends on the potential between the surface and the electrolyte solution. Electrokinetic measurement is one way to study complex surface chemical exchange processes at the clay mineral surface/liquid interface. All electrokinetic phenomena are related to the development of electrical double layer at the particle/electrolyte interface. The zeta potential is defined as the potential of shear plane of the particle when it moves in liquid [28]. ZP is one of the very important electrokinetic properties of minerals. The results of ZP studies suggested that the mucilages are ionic in nature and attributed to the presence of uronic acids as similar to what was previously reported by Mohammadifar et al. for gum tragacanth which is uronic acid-rich gum [29]. Polarity of mucilage may be due to the presence of –OH group, which is readily available for the formation of hydrogen bonds on the interface of reinforced systems. However, plant mucilages are covered with pectin and waxy substances, thus hindering the hydroxyl groups (–OH) from reacting with polar matrices and deteriorate adhesion, but polar structure is only responsible for hydrophilic behavior of mucilage. Further, this indicates that mucilages swell strongly in an aqueous environment. For high adhesion forces, it is necessary to have polar functional groups, but swelling processes should be prevented. According to Kanamaru, the amount of zeta potential should decrease with time for an advancing swelling process. This process is completed, when the value of ZP remains constant. The extent of mucus adsorption is proportional to the absolute values of the positive zeta potential of mucilage and negative “ZP” of mucus glycoprotein [30]. Shashikant reported that the significant difference in ZP of a polymer may be obtained due to change in environment, that is, particles decomposition which yield small charge and surface properties of particles [31]. Further, the electrophoretic mobility of suspended particle depends on ZP. It was concluded that conductivity of any solution is a function of electrolytes or ions and charge in ionic concentration is known to increase conductivity. The conductivity and dielectric constant often increase with thermal and electrical stress. These changes therefore are indicative of decomposition of material to yield some smaller molecules. This also finds support in the fact that there is concomitant shift in UV and IR spectra [31]. Taking account of earlier studies it can be believed that the ZP caused huge impact on properties of mucilage.

### 4.8. Powder X-Ray Diffraction Pattern

The powder X-ray diffraction is a useful method for investigating the arrangement of atom and molecules within the material. If there is an orderly arrangement of substructure within the material with repeat distances of a similar magnitude to the wavelength of light used, interference patterns are produced, and such patterns provide information on geometry of polymer structure. Powder XRD analysis of mucilage sample showed no characteristic sharp peaks in the spectrum and indicated that the mucilage is completely amorphous in nature. Natural gums, such as Arabic, guar gum, and karaya gum, also show amorphous nature [32].

### 4.9. Fourier Transform Analysis

FTIR spectrometry has been extensively applied to characterize the polymer's molecular and material structure. Characterization using FTIR spectroscopy often results in the identification of functional groups and the modes of their attachment to polymer backbone [33]. The FTIR spectra exhibit the typical bands and peak characteristic for mucilage. The broad band occurring at 3500–3200 cm^−1^ results from the presence of hydroxyl (–OH) groups. The peak obtained at 2885–2705 cm^−1^ results from stretching modes of the C–H bonds of methyl groups (–CH_3_). Natural gums usually contain fractions of sugar acid units which would usually impart a weakly anionic character to the gum macromolecule [23]. Absorption bands around 1618 and 1430 cm^−1^ are typical of carboxylate groups of the galacturonic acid residues as reported by Okafor et al. [34, 35]. The region between 1500 and 1800 cm^−1^ is typically used to detect presence of carboxylic groups. Also, absorption peaks at 1740 cm^−1^ and 1258 cm^−1^ are typical of acetyl groups [35]. The wave numbers between 800 and 1200 cm^−1^ represents the finger print region for carbohydrates [36].

### 4.10. 1D Nuclear Magnetic Resonance

NMR is the most powerful tool for the study of microstructure and chain configuration of polymers, both in solution and in solid state. The importance of NMR as a technique is from the fact that NMR signals can be assigned to specific atoms along the polymer backbone and side chains. The ^1^H NMR spectrum for mucilage showed few singlet at high field (d 1.1785 ppm (s), d 1.9559 ppm (s)), which is related to the environments of methyl groups of rhamnose and the protons linked to C-6 (d 3.65, d 3.70 ppm) and C-4 of galactose (d 3.98, 4.28 ppm), respectively, and this suggests the existence of different galactose derivatives [37]. The anomeric protons have been assigned to *β*-sugar residues (d 4.92–4.96 ppm) and the *α*-sugar residue (d 5.1–5.3 ppm), as reported earlier by Agrawal [38]. The two closely neighbored signals observed in the ^1^H NMR spectrum of mucilage (d 4.02 and d 3.84 ppm) were assigned to H-1 of *α*-glucose [39]. The ^1^H NMR spectrum showed that crowded narrow region between 3 to 5 ppm is typical of polysaccharides and confirms the presence of many similar sugar residues [35]. The signals between 3.1 and 4.3 ppm can be assigned to nonanomeric protons (H_2_–H_6_), while signals between 4.3 and 4.8, and 4.9 and 5.5 ppm arise from *α*-anomeric and *β*-anomeric protons, respectively. ^13^C NMR spectra of mucilages gave line widths which are typical of an amorphous natural polymer with broad band signals. There are resonance spectra due to the methyl group of rhamnose (d 16.67 ppm) as reported earlier by Martínez et al. [40].

### 4.11. Sugar Composition by Paper Chromatography

Plant gums and mucilages are an important group of plant constituents with pharmaceutical and technical uses. The separation and sensitive determination of various carbohydrates are currently of great interest in nutrition, medical cell biology, and biotechnology research. Carbohydrates are difficult to analyze because they are very polar compounds, exhibit similar structural characteristics, and do not have a suitable chromophore. The paper chromatography analysis was performed in order to check the available carbohydrates in mucilage. The result obtained from 1D NMR, FTIR, and paper chromatography studies indicated the presence of arabinose, fructose, mannose, rhamnose, xylose, and glucose. The result obtained from the study indicated similar *R*_*f*_ values to the standard available data for sugars. According to Lee, arabinose is reported to be a free, but minor, sugar component of onions, grapes, strawberries, commercial beer, corn, and alfalfa [41].

## 5. Conclusions

*Manilkara zapota* (Linn.) P. Royen syn. seeds mucilage is a novel polysaccharide gum with little published information on its characterization. It is a natural, biodegradable, nontoxic material and requires lower production cost. The overall findings of the study indicated that mucilage isolated from the seeds have an inherent property which can be used in various dosage forms on modification. The PXRD and SEM revealed that mucilage is amorphous in nature which indicated that wet granulation technology will be more suitable for dosage formulation. Significant mineral and elemental composition was obtained. The thermal stability study by DSC, DTA, and TGA showed that mucilage had good thermal stability. FTIR, solid 1D ^1^H, and C^13^ NMR and paper chromatography confirmed presence of nonreducing sugars. All these results demonstrate that mucilage is a useful pharmaceutical aid and can be used for effectively controlling the release of drugs from the designed matrix systems.

## Figures and Tables

**Table 1:** Gel permeation chromatography characterization of mucilage.

Polymer	*M*_*n*_	*M*_*w*_	*M*_*p*_	*M*_*z*_	*M*_*z*_ + 1	Polydispersity	*M*_*w*_/*M*_*n*_
Mucilage	58953	379180	50662	1724635	2903837	6.431905	6.43190

**Table 2:** Thermogravimetry analysis of mucilage.

Mucilage	Decomposition stage	Temperature range (°C)	DTG peak (°C)	Enthalpy (J/g)	Heat change (µVs/mg)	% weight loss
Mucilage	1	178.6–359.7	350.3	315.8729	138.4354	41.17
	2	359.7–600.6	614.4	3624.787	1082.215	30.06

**Table 3:** Elemental composition and heavy metal characterization of mucilage.

Polymer	C (%)	H (%)	N (%)	W_C/N_	S (mg/kg)	Arsenic	Lead	Cadmium	Mercury
Mucilage	80.9	10.1	1.58	51.202	512	ND	ND	ND	ND

ND: not detectable.
